# Robot- vs Video-Assisted Thoracoscopic Lobectomy for Early Lung Cancer

**DOI:** 10.1093/jncics/pkaa031

**Published:** 2020-04-15

**Authors:** Vignesh Raman, John K Christopher, Oliver K Jawitz, Jacob A Klapper

**Affiliations:** Division of Cardiovascular and Thoracic Surgery, Department of Surgery, Duke University Medical Center, Durham, NC, USA

Robot-assisted thoracoscopic surgery (RATS) is increasingly considered an alternative to video-assisted thoracoscopic surgery (VATS) and thoracotomy for patients with resectable non-small cell lung cancer (NSCLC). RATS has several potential advantages compared with VATS, including increased intraoperative rotational capacity, three-dimensional visualization, a shorter hospitalization, and improved postoperative analgesia, although these have not been demonstrated uniformly or in prospective, randomized trials (1–3). However, RATS has been associated with increased costs, and early reports revealed a potentially increased risk of cardiovascular complications compared with VATS (4,5). Further, the comparative oncologic effectiveness of RATS compared with VATS has not been demonstrated in prospective trials, although single institution and registry analyses have demonstrated similar survival in patients undergoing lobectomy with either approach (6,7).

In this issue of the Journal, building on previous observational studies, Dr Cui and colleagues (8) used the National Cancer Database (NCDB) to examine the comparative survival of 18 908 patients undergoing RATS vs VATS lobectomy for stage I NSCLC. Using an intention-to-treat design, the authors found that RATS lobectomy was associated with worse overall survival compared with VATS in patients with tumors no more than 20 mm even after adjustment for age, number of comorbidities, histology, hospital surgical volume, and other variables. This disparity in survival persisted after 1:1 and N:1 propensity score-matching as well. The authors demonstrated worse adjusted survival at landmark times 12, 18, and 24 months following surgery as well as in patients who did not undergo conversion to thoracotomy. However, in patients with tumors larger than 20 mm, the authors did not find a difference in survival between RATS and VATS.

Although several previous studies, including in the NCDB, have found similar overall survival between patients undergoing VATS and RATS lobectomy, this is the first study that identifies a population of patients, based on tumor size, who are at a higher risk for mortality associated with RATS lobectomy. The authors describe a well-designed and methodologically sound retrospective cohort analysis. They found a significant interaction between tumor size and type of lobectomy in a multivariable Cox model of the overall cohort of patients, leading them to stratify patients based on tumor size for subsequent analyses, ultimately demonstrating that tumor size mediates the relationship between approach to surgery (VATS vs RATS) and survival in the cohort studied. Their multivariable Cox models appropriately adjusted for all relevant prognostic variables available in the NCDB, especially age, histology, grade, type of treatment center, year of diagnosis, and center surgical volume. Further, the authors demonstrate that the interaction between tumor size and approach to surgery is independent from center surgical volume, which often confounds survival analyses in observational studies of surgery. The authors also used conditional landmark analyses to show that RATS is associated with worse survival compared with VATS when follow-up was started 12, 18, and 24 months from surgery in patients with smaller tumors.

The finding that RATS lobectomy is associated with worse survival compared with VATS lobectomy in patients with tumors smaller than 20 mm is provocative, and the authors do not proffer an explanation in their manuscript. The persistence of this finding in landmark analyses suggests that the survival disparity is not merely attributable to perioperative events. From a technical perspective, we cannot imagine a plausible explanation for RATS being associated with worse long-term survival compared with VATS for smaller tumors, because both approaches are likely to effectively enable complete resection via lobectomy. We considered the possibility that in this intention-to-treat analysis, the unmeasured difference may have been the propensity for pathologic nodal upstaging in the 2 groups, which the authors do not report in their manuscript. For instance, if RATS patients were more likely to be upstaged, they would be more likely to experience worse survival compared with VATS patients. However, a propensity score-matched NCDB analysis revealed similar incidence of nodal upstaging in VATS and RATS patients, which has been corroborated in other data sets as well (1,3,7).

In a multivariable logistic regression in the 2004–2015 version of the NCDB, our group also found that RATS was associated with similar odds of pathologic nodal upstaging compared with VATS lobectomy in 11 747 patients with clinical stage I NSCLC and tumor no more than 20 mm (adjusted odds ratio [OR] = 0.91, 95% confidence interval [CI] = 0.78 to 1.07). Given the interaction between tumor size and approach to lobectomy, we also wondered if there were other interactions, including three-way interactions, in the group of patients with smaller tumors. We found persistent interactions between histology and approach to surgery and center volume and approach to surgery in this group of 11 747 patients, but these interactions did not substantially change the treatment effect associated with RATS compared with VATS lobectomy in multivariable Cox regression. Our best hypothesis is that the survival difference observed between these groups is attributable to selection bias and unmeasured confounders.

The Society of Thoracic Surgeons database analysis of 12 378 VATS and 1220 RATS patients with clinical stage I–II NSCLC offers the best insight to what these confounders may be (1). In this study, patients who underwent RATS were more likely to be less physically active, have a higher body mass index, and have coronary artery disease compared with those who underwent VATS lobectomy. Although the Society of Thoracic Surgeons registry does not capture mid- or long-term survival, these differences in baseline characteristics may be expected to worsen the survival of patients who undergo RATS rather than VATS lobectomy, which may explain the finding in the current study under discussion. Similarly, the NCDB does not capture measures of frailty, malnutrition, and preoperative pulmonary function. Further, in the absence of information about cancer-related survival, it is difficult to attribute observations about overall survival to an oncologic etiology, which forms the crux of this study and others examining different minimally invasive approaches for cancer.

In this NCDB analysis, Dr Cui and colleagues (8) report a novel finding that in patients with clinical stage I NSCLC and tumors no more than 20 mm, RATS is associated with substantially worse overall survival compared with VATS lobectomy, although survival is similar between the groups in patients with larger tumors. Although unmeasured confounding is the most likely explanation for these findings, it is important that this finding be tested in other registries and potentially in a prospective trial in order for us to provide the best care possible for patients with early lung cancer.

## Notes

**Role of the funder:** This work received no direct funding.

**Conflicts of interest:** The authors have no conflicts of interest to disclose.
